# Editorial: Impact of digital addiction on mental health and comorbidities in older adults

**DOI:** 10.3389/fnbeh.2026.1988680

**Published:** 2026-09-28

**Authors:** Sabrina Molinaro, Gergely Feher, Roberto Truzoli

**Affiliations:** 1 Institute of Clinical Physiology, National Research Council (CNR), Pisa, Italy; 2 Pecsi Tudomanyegyetem Altalanos Orvostudomanyi Kar, Pécs, Hungary; 3 University of Milan Department of Biomedical and Clinical Sciences, Milan, Italy

**Keywords:** digital addiction, healthy aging, mental health, older adults, problematic digital technology use

Digital technologies have become an integral part of everyday life across the lifespan. Yet research on problematic internet and smartphone use has traditionally concentrated on adolescents and young adults, leaving substantially less evidence on later life. This imbalance has become increasingly difficult to justify. Population aging is occurring alongside the progressive integration of digital technologies into everyday activities, social relationships, healthcare, and independent living (Kebede et al., 2022). Older adults are therefore not peripheral to the digital transformation, although their patterns of use, motivations, and vulnerabilities may differ substantially from those observed in younger generations.

This was the starting point of this Research Topic. Its contributions suggest, however, that extending the study of digital addiction to older populations requires more than simply applying concepts and instruments developed for younger people to a different age group. Aging changes the context in which technology is used. Retirement, changes in social networks, loneliness, physical limitations, health conditions, and cognitive changes may influence both the reasons for engaging with digital technologies and the circumstances under which such engagement becomes problematic. Existing evidence indicates that internet addiction among older adults may emerge from the interaction of individual, family, social, and technological factors (Wang et al., 2024), rather than from a single dimension of excessive exposure.

Perhaps the first distinction that needs to be made is therefore between digital engagement and problematic digital use. This distinction emerges clearly from the contributions to this collection. Hu et al. found that internet use among rural older adults was associated with better mental health, partly through greater social participation, suggesting that digital technologies may support connectedness and inclusion rather than simply represent a behavioral risk. This finding resonates with previous evidence showing that smartphone use among community-dwelling older adults is not inherently associated with poorer health (Bertocchi et al., 2022). Prior evidence has also shown that smartphone use patterns and digital literacy influence the type and risk of problematic smartphone use among older adults (Jeong and Bae, 2022), while digitalization increasingly reshapes social relationships and everyday support networks in old age (Hülür and Macdonald, 2020). Digital inclusion may therefore constitute a resource for healthy aging; the relevant question is when and under what conditions engagement shifts from adaptive to dysfunctional.

The studies collected here suggest that this transition cannot be understood through screen time alone. Problematic internet use appears embedded in a broader constellation of psychological and social vulnerabilities. Across different stages of the COVID-19 pandemic, Chen X.-M. et al. showed that its relationship with psychological distress among older teachers involved fear, frustration of psychological needs, and occupational burnout. In other contexts, problematic use was associated with depression, insomnia, emotional exhaustion, and poorer quality of life among recreational esports players studied by Feher et al., while the study by Radvanyi et al. extended the picture beyond mental health by examining the relationship between internet use and primary headache severity. Previous research in adults over 65 similarly links addictive smartphone use with depression, anxiety, and poorer sleep quality (Karaş et al., 2023).

Taken together, these findings point toward a continuum in which digital behavior interacts with wellbeing, social resources, occupational circumstances, sleep, and physical health. This interpretation is reinforced by Gui et al., who used interpretable machine learning to examine the co-occurrence of internet addiction and maladaptive cognitive emotion regulation among community-dwelling older adults. General wellbeing emerged as the strongest predictor, together with educational attainment and support utilization. Rather than representing an isolated behavior, problematic digital use may thus be one expression of a broader configuration of vulnerability.

This perspective also changes how prevention should be conceptualized. If problematic use develops within a wider psychosocial context, prevention cannot consist solely of limiting access or reducing time spent online. The longitudinal evidence provided by Chen B. et al. is particularly informative in this respect: positive exercise experiences predicted lower subsequent mobile phone addiction tendencies among older adults. Promoting alternative sources of reward, physical activity, participation, and social connection may therefore be as important as targeting digital behavior itself.

At the same time, responsibility cannot be placed exclusively on the individual. One of the most relevant developments emerging from this collection is the shift from studying the vulnerable user to studying the potentially addictive digital environment. Li et al. found that personalized recommendations, interactive incentives, economic rewards, and goal-feedback mechanisms were associated with digital addiction among older adults, highlighting how algorithmic design may contribute to maintaining engagement. This is especially relevant in an aging population, where differences in digital literacy, cognitive resources, or familiarity with persuasive technologies may affect the ability to recognize and regulate such mechanisms.

Generative artificial intelligence further complicates this relationship. The review by Vasiliu discusses emotional attachment, anthropomorphism, immediate reinforcement, and parasocial interactions as possible mechanisms underlying problematic engagement with generative AI chatbots. Yet the evidence remains preliminary, and the review appropriately cautions against pre-maturely transforming intensive engagement into a new diagnostic category. Across these different digital environments, loss of control, functional impairment, and psychological distress appear more meaningful than frequency of use alone.

This distinction becomes particularly important when considering cognitive aging. Cognitive functioning is one of the areas in which older adults differ most fundamentally from the populations on which much of the digital addiction literature has been built. Age-related changes in attention, memory, and executive functioning could theoretically influence susceptibility to persuasive digital environments, while problematic patterns of engagement could themselves interact with cognitive health. Nevertheless, the scoping review by Jiang et al. highlights how little we currently know about this relationship. Across 22 studies of smartphone-related addictive behaviors in adults aged 60 years and older, evidence was substantially stronger for sleep, depression, anxiety, and loneliness than for cognition, and the available cognitive evidence was insufficient to support conclusions about cognitive decline. Moreover, 18 of the 22 studies were cross-sectional, further limiting causal interpretation.

This absence of evidence is itself important. Digital technology cannot simply be assumed to be detrimental to cognitive health. Smartphones and other digital tools may support autonomy, communication, access to information, and everyday functioning, and previous evidence suggests differences in cognitive performance between older smartphone users and non-users (Bertocchi et al., 2022). The critical question is therefore not whether technology is good or bad for the aging brain, but which patterns of use are beneficial, which become problematic, for whom, and at what point in the aging process. Longitudinal research is needed to determine whether problematic digital behaviors influence cognitive trajectories, whether emerging cognitive vulnerability increases susceptibility to loss of control and algorithmic persuasion, or whether these relationships are bidirectional.

The evidence assembled in this Research Topic therefore argues for a concept of healthy digital aging based on balance rather than restriction. Digital participation can support autonomy, social connection, and inclusion, while problematic engagement may coexist with psychological distress, sleep disturbances, reduced wellbeing, and other health problems. As digital environments evolve from smartphones and social media toward personalized algorithms and generative AI, understanding where this boundary lies will become increasingly important.

Moving the field forward will require age-sensitive measures, longitudinal and cross-cultural studies, and greater integration of mental, physical, social, and cognitive health. Most importantly, research should move beyond screen time and individual responsibility toward understanding the interaction between the person, their social and health context, and the digital environment itself. Only then will it be possible to distinguish digital engagement that supports healthy aging from patterns that genuinely compromise autonomy, functioning, and wellbeing.
